# A Direct Investment Method of Closed Two-Piece Hollow Bulb Obturator

**DOI:** 10.1155/2013/326530

**Published:** 2013-06-27

**Authors:** Suryakant C. Deogade, Sneha S. Mantri, Dinesh Naitam, Gunjan Dube, Pushkar Gupta, Ashish Dewangan

**Affiliations:** ^1^Department of Prosthodontics, Hitkarini Dental College & Hospital, Jabalpur, Madhya Pradesh 482001, India; ^2^Department of Prosthodontics, Rungta College of Dental Sciences & Research, Bhilai, Chhattisgarh 490024, India; ^3^Department of Oral Surgery, Hitkarini Dental College & Hospital, Jabalpur, Madhya Pradesh 482001, India

## Abstract

Maxillary defects occur due to surgical treatment of benign and malignant tumors, congenital malformation, and trauma. Prosthetic rehabilitation in such patients is influenced by the size and location of the defect. The most common of all intraoral defects are seen in the maxilla, in the form of an opening into the maxillary sinus and nasopharynx. These defects create disabilities in speech, deglutition, and mastication. The prosthesis which closes such an opening and recreates the functional separation of the oral cavity and sinus and nasal cavities is referred to as an obturator. Numerous techniques of hollow bulb fabrication have been mentioned in the literature from time to time. But there are only a few methods for bulb fabrication in two-piece obturator. This technique describes a direct investment method of waxed-up closed hollow bulb two-piece obturator.

## 1. Introduction

Maxillofacial prosthetics is the branch of prosthodontics concerned with the restoration and/or replacement of the stomatognathic and craniofacial structures with prostheses that may or may not be removed on a regular or elective basis [1]. The most common of all intraoral defects are seen in the maxilla, and it may include hard and soft palate, maxillary sinus, floor of the nasal cavity, and the alveolar ridges [2]. These defects may occur due to surgical resection of benign and malignant tumors, congenital malformation, and trauma [3].

Maxillary intraoral defects due to surgical resection create an open link between the oral and nasal cavities causing difficulty in deglutition, speech, and an unaesthetic appearance. It also results in psychological trauma to the patient [4]. The goals of prosthetic rehabilitation for such patients are to fabricate obturators which separate the nasal and oral cavity and improve deglutition, speech, mastication, and esthetics [2, 3]. 

An obturator (Latin: obturare, to stop up) is a disc or plate, natural or artificial, which closes an opening or defect of the maxilla as a result of a cleft palate or partial or total removal of the maxilla for a tumor mass [5]. There are numerous techniques described in the literature for the fabrication of open and closed hollow obturators [6–14]. Most of these techniques have their own limitations, such as multiple processing steps while fabrication [15, 16]. All these types of obturators reduce the weight of the prosthesis while properly extending into the defect. Open hollow obturators tend to accumulate nasal secretions leading to odour and added weight [4, 17, 18]. Hence, these require frequent cleaning. Also, it is difficult to polish and clean the internal surface from saliva, mucous crusts, and food accumulation. Where as in closed hollow bulb obturators, pooling of moisture is eliminated. Also, it extends superiorly into the defect, thereby reducing the air space [19].

In this case report, the patient was having severely restricted mouth opening, which required the fabrication of two-piece obturator prosthesis. The fabrication method of the two-piece closed hollow bulb obturator with direct investment method is described in this report.

## 2. Case Report

**Figure 1:**
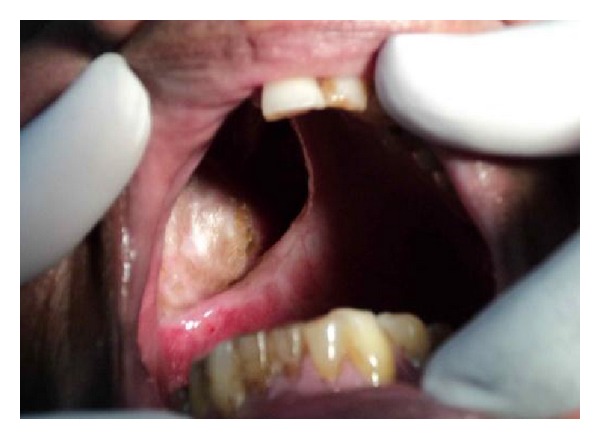
Intraoral defect.

A 70-year old-man was reported to the Department of Prosthodontics, Hitkarini Dental College and Hospital, Jabalpur, India, with a complain of difficulty in speech, deglutition, and mastication. The patient was operated on with maxillectomy to the right side to treat squamous cell carcinoma about four months back. He was wearing on interim obturator prosthesis since four months and demanded a definitive prosthesis. The mouth opening of the patient was severely restricted due to postsurgical scar formation and radiation therapy (Figure 1). Hence, the decision was taken to fabricate a two-piece obturator prosthesis where the obturator was fabricated separately by direct investment technique. Also the magnets were chosen as a method of retention between the obturator and the basic prosthesis. However, only the technique of an obturator fabrication is described.

## 3. Procedure

**Figure 2:**
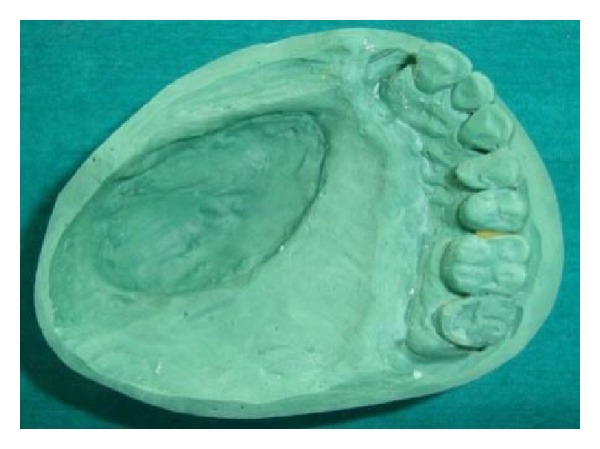
Definitive cast.

Preliminary impression of maxillary arch along with defect was made using irreversible hydrocolloid (Dentalgin; Prime Dental Products, Mumbai, India), and the cast was poured with type II gypsum material. After that, the special tray was fabricated with an autopolymerizing acrylic resin (DPI cold cure; Dental Products of India, Mumbai, India), and the definitive impression was made with medium viscosity poly (vinyl siloxane) impression material (Reprosil; DENTSPLY DeTrey GmbH, Konstanz, Germany). All the conventional prosthodontic protocols of boxing and pouring the impression were applied with type III gypsum material (Kalstone; Kalabhai Karson, Mumbai, India) to create a definitive cast (Figure 2). An additional cast was duplicated for the fabrication of closed hollow bulb separately.

The basic prosthesis was fabricated on the master cast by a conventional manner. The procedure of hollow bulb fabrication on the duplicated cast is as follows.

**Figure 3:**
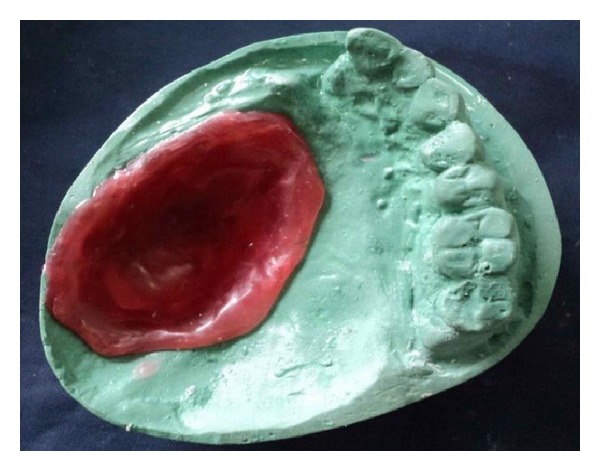
Adaptation of a layer of wax.

**Figure 4:**
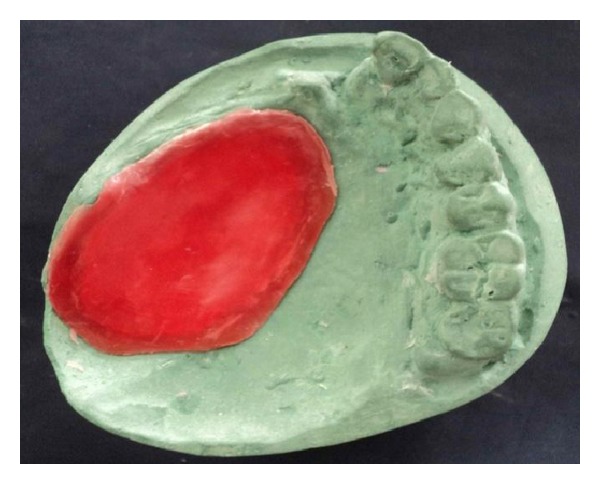
Sealed wax lid.

**Figure 5:**
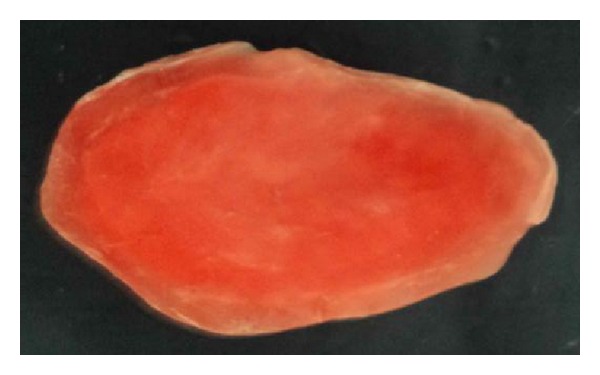
Waxed-up bulb.

**Figure 6:**
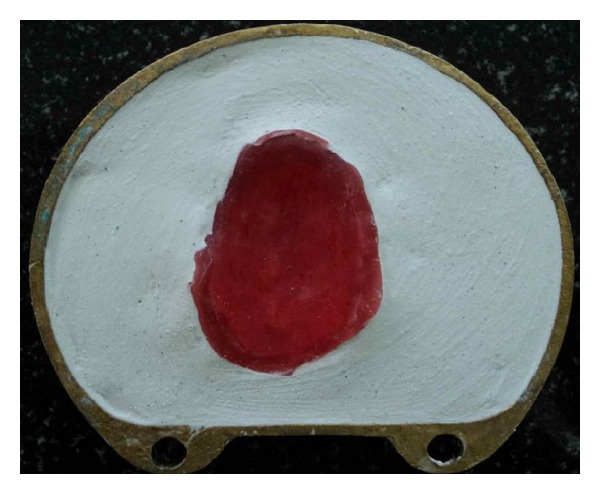
Flasking of waxed-up bulb.

**Figure 7:**
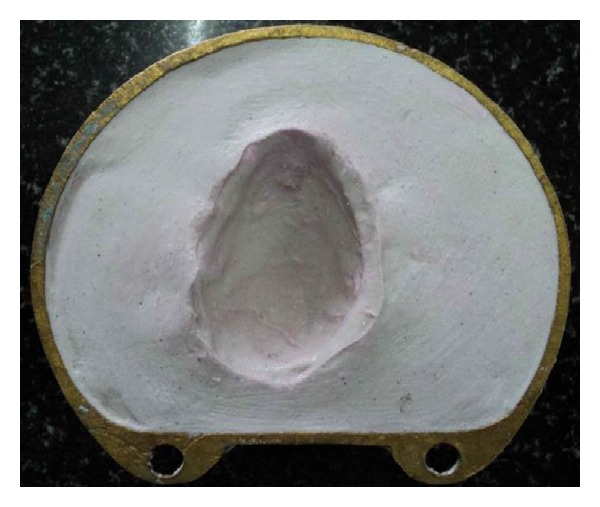
Dewaxing of waxed-up bulb.

**Figure 8:**
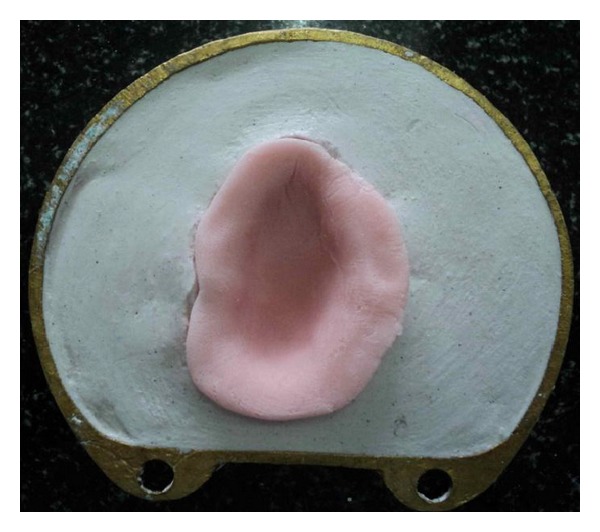
Packing with heat-cured PMMA.

**Figure 9:**
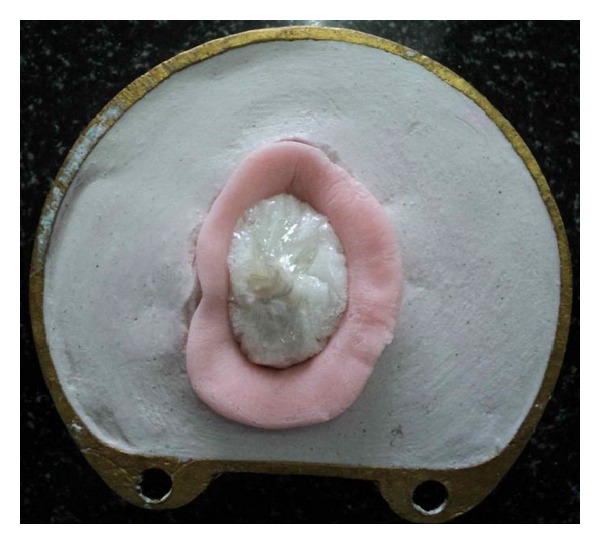
A pouch of packed salt.

**Figure 10:**
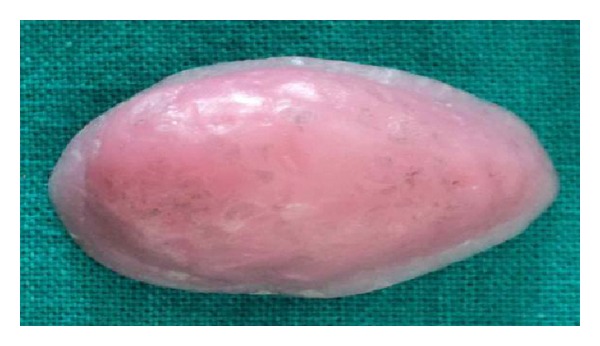
Defect side view of bulb.

**Figure 11:**
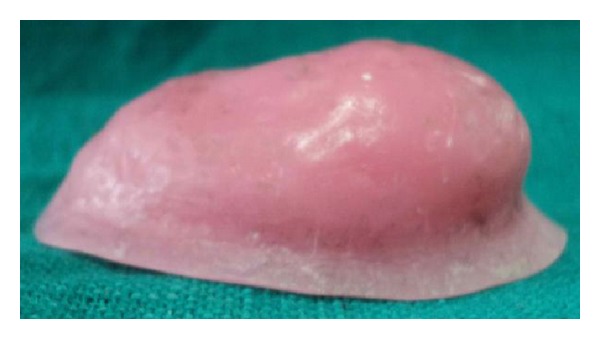
Lateral view of bulb.

**Figure 12:**
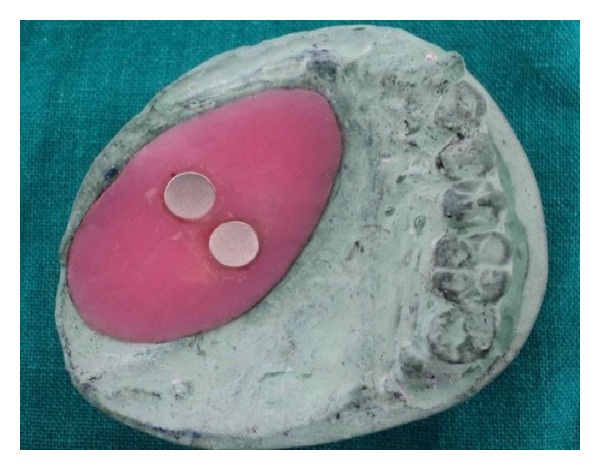
Finished bulb with magnets.

**Figure 13:**
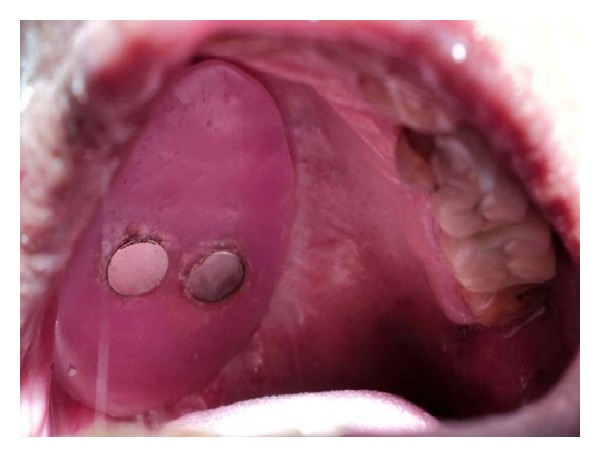
Bulb in patient's mouth.

**Figure 14:**
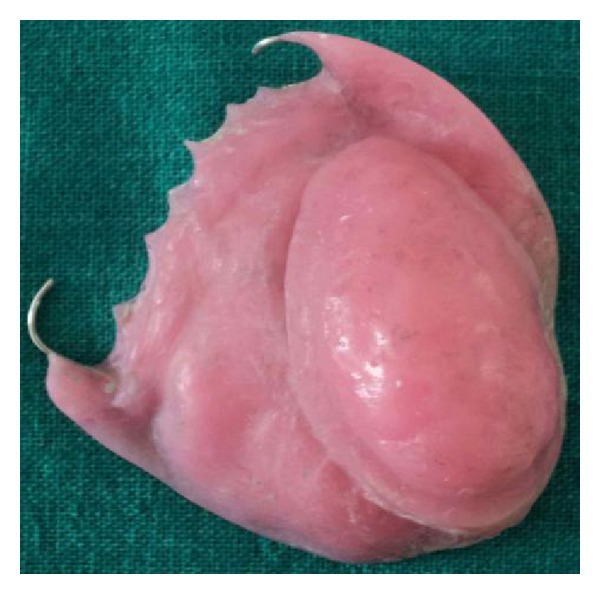
Bulb with prosthesis.

**Figure 15:**
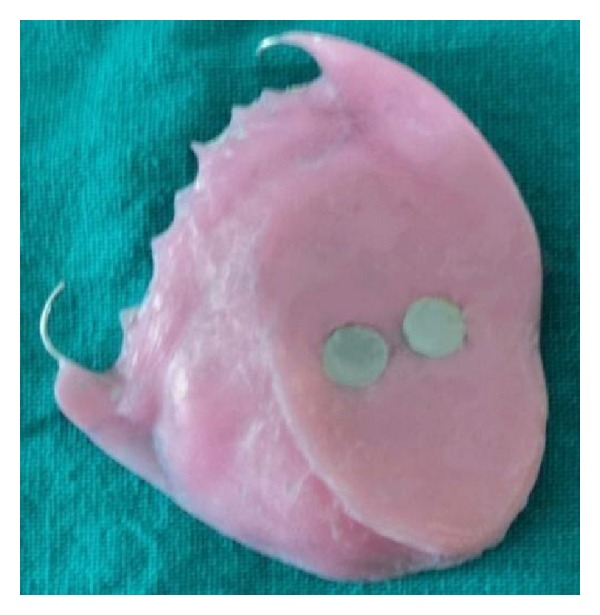
Tissue surface with magnets.

**Figure 16:**
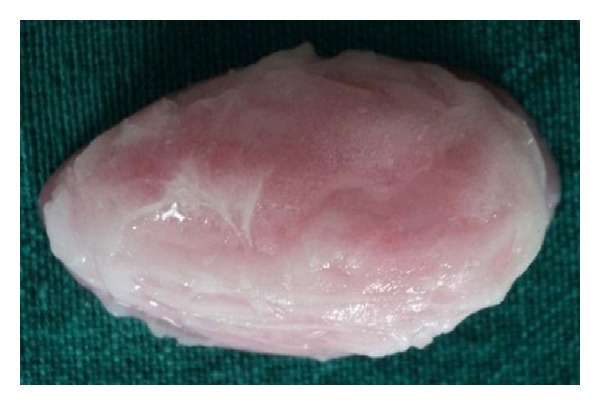
Bulb with soft liner.

**Figure 17:**
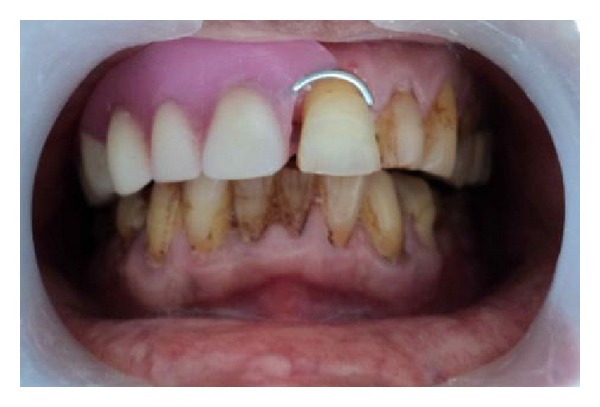
Prosthesis in patient's mouth.

1. Undercuts in the defect area were blocked out with the plaster of Paris.
2. A 2 mm thick base plate wax was adapted over the defect area of the duplicated cast (Figure 3).
3. Then, modeling clay was packed into the defect part, and a tinfoil was kept over it. A 4 mm thick wax lid was adapted on the tinfoil.
4. After that, already fabricated waxed-up denture was seated over it to maintain the palatal contour. The waxed-up denture was then removed, and the wax lid was sealed on it after removing the clay (Figure 4).
5. The waxed bulb was then removed carefully, and flasking and dewaxing procedures were completed in a conventional manner (Figures 5, 6, and 7). The mold space was packed with heat-polymerizing acrylic material (DPI, Mumbai, India) (Figure 8).
6. During packing of material, a pouch of salt was used to hollow the bulb by lost salt technique (Figure 9). Curing procedures were performed according to the manufacturer's instructions.
7. The cured bulb was then retrieved after deflasking, and the salt was removed after drilling 2 mm holes in the lid portion. The holes were drilled at the future location of magnet attachment.
8. Then, a pair of commercially available closed-field magnets (Cobalt-Samarium, Ambika Corporation, New Delhi, India), 5 mm in diameter and 2 mm in thickness, was positioned with the help of autopolymerizing acrylic resin, and finishing and polishing were carried out in the conventional manner.
9. Care was taken not to over thin the bulb, because perforation may occur during the finishing and polishing of the prosthesis.
10. The finished and polished bulb was then checked on the master cast for its proper fit (Figures 10, 11, and 12). After performing adjustments, the bulb was checked in patient's mouth (Figure 13).
11. Then the bulb was kept on the cast, and a marking was done on the top portion of magnets with a copying pencil. The finished and polished prosthesis was then tried to seat on the cast. The marks transferred on the tissue surface of the prosthesis determined the position of the countermagnets.
12. A provision of space was done in the tissue surface of the prosthesis, and the countermagnets were kept over the magnets of the bulb. These were fixed to the prosthesis with the help of self-cure acrylic resin (Figures 14 and 15). After that, a temporary liner (PermaSoft, Soft Denture Liner, DENTSPLY, Austenal) was applied and placed in patient's mouth (Figure 16). After one week, it was replaced with the permanent liner. The patient was educated about positioning, removal, and cleaning of the obturator prosthesis. Patient's demands were fulfilled (Figure 17).

## 4. Discussion

The design and fabrication of the bulb portion is the most important and demanding task for the success of an obturator prosthesis [5, 20]. Numerous articles are mentioned in the literature describing techniques for the fabrication of hollow obturators to lighten the weight of the prostheses. But only few describe the closed hollow bulb in two-pieced obturator prosthesis. A two-piece obturator is given only when the maxillary defect is large with more undercuts, and when the prosthesis is large such that it interferes with its insertion. It is also preferred in those patients who show severely restricted mouth opening due to postsurgical scar formation and radiation therapy [5, 21, 22].

The technique described in this procedure can be applied to complete or partially edentulous patients who have had severely restricted mouth opening. In severe trismus, it becomes very difficult for a patient to insert and remove the prosthesis in a single piece. Hence, it is always better to construct a two-piece obturator for an easy and convenient insertion and removal of prosthesis by the patient. The technique described here utilizes a duplicated cast for the fabrication of bulb separately. The carving and finishing of the waxed-up bulb does not destroy the master cast in this way. Even, the minor adjustment can be done after finishing and polishing the cured bulb. The attachment of retentive means like magnets becomes easy and convenient by this technique. Also, hollowing of the obturator becomes very easy and straightforward. Furthermore, the future relining with permanent soft liner material becomes an easy job.

This technique really works in two-piece obturator due to its ease of fabrication. Even the strength of the bulb is maximized through heat processing of the acrylic resin while minimizing porosity and increasing durability. The thickness of the bulb is easily controlled, which allows for a lightweight prosthesis. The best part of this technique is that the accuracy is assured.

## 5. Conclusion

The hollow bulb obturator given to the patient rehabilitated his function by improving masticatory efficiency and phonetics by adding resonance to the voice. Thus, it improved the clarity of speech and the esthetics of the patient. It improved the comfort of the patient by decreasing the weight of the prosthesis. The two-piece prosthesis was easily tolerated by the patient and made convenient in terms of easy insertion and removal of the prosthesis.

Simplicity of fabrication and assurance of accuracy are the basic advantages of this technique. The technique provides control of the thickness of the hollow bulb, subsequently reducing the overall weight. Patient's functional and esthetic requirements were fulfilled satisfactorily. It improved the morale of the patient greatly.
